# Alterations in Cellular Iron Metabolism Provide More Therapeutic Opportunities for Cancer

**DOI:** 10.3390/ijms19051545

**Published:** 2018-05-22

**Authors:** Liangfu Zhou, Bin Zhao, Lixiu Zhang, Shenghang Wang, Dandan Dong, Huanhuan Lv, Peng Shang

**Affiliations:** 1 School of Life Science, Northwestern Polytechnical University, Xi’an 710072, China; zlf19900919@mail.nwpu.edu.cn (L.Z.); zhao000bin@mail.nwpu.edu.cn (B.Z.); zlx1992@mail.nwpu.edu.cn (L.Z.); wangshenghang@mail.nwpu.edu.cn (S.W.); ddd2013@mail.nwpu.edu.cn (D.D.); lvhh2017@nwpu.edu.cn (H.L.); 2 Research & Development Institute in Shenzhen, Northwestern Polytechnical University, Shenzhen 518057, China; 3 Key Laboratory for Space Bioscience and Biotechnology, Institute of Special Environmental Biophysics, Northwestern Polytechnical University, Xi’an 710072, China

**Keywords:** iron metabolism, cancer, iron chelators, targeted therapy, redox homeostasis

## Abstract

Iron is an essential element for the growth and proliferation of cells. Cellular iron uptake, storage, utilization and export are tightly regulated to maintain iron homeostasis. However, cellular iron metabolism pathways are disturbed in most cancer cells. To maintain rapid growth and proliferation, cancer cells acquire large amounts of iron by altering expression of iron metabolism- related proteins. In this paper, normal cellular iron metabolism and the alterations of iron metabolic pathways in cancer cells were summarized. Therapeutic strategies based on targeting the altered iron metabolism were also discussed and disrupting redox homeostasis by intracellular high levels of iron provides new insight for cancer therapy. Altered iron metabolism constitutes a promising therapeutic target for cancer therapy.

## 1. Introduction

Iron is a vital trace element for most living creatures on Earth. The human body contains 3–5 g iron which is mainly distributed in red cells, bone marrow, muscle, liver and macrophages [1]. The content of body iron is maintained at a stable level. Duodenal enterocytes can absorb 1–2 mg iron from a daily diet, and 20–25 mg iron is recycled from aged red blood cells [2]. Humans have no physiologic pathways for iron excretion except through shedding of mucosal and skin cells and blood loss [3]. Iron is essential for cell growth, proliferation and differentiation. Heme-iron serves as a cofactor for hemoglobin and myoglobin involved in many important physiological processes including oxygen binding and transport, and oxygen metabolism; non-heme iron is the active center of many important enzymes involved in DNA synthesis and cell cycle [4].

The biological activity of iron lies in its ability to accept or donate electrons. The efficient electron transferring properties enable iron to participate in many reactions, such as the Fenton reaction. Evidences suggest that excess iron in the body may lead to increased risk of cancer, which is partly due to free radicals produced by Fenton reaction which further damages DNA [5]. Iron also plays a crucial role in promoting cancer cell proliferation, because iron contributes to DNA synthesis. Therefore, the high requirement of iron is prevalent in cancer cells. Mounting evidence indicates that changes in iron metabolism-related proteins contribute to tumor initiation and growth. Meanwhile, many clinical studies have provided new insights into cancer treatment by altering iron metabolism. Herein, we review the iron metabolism in normal cells and changes of it in cancer cells, and potential iron metabolism-targeted therapeutics for cancer.

## 2. Normal Cellular Iron Metabolism

**Figure 1:**
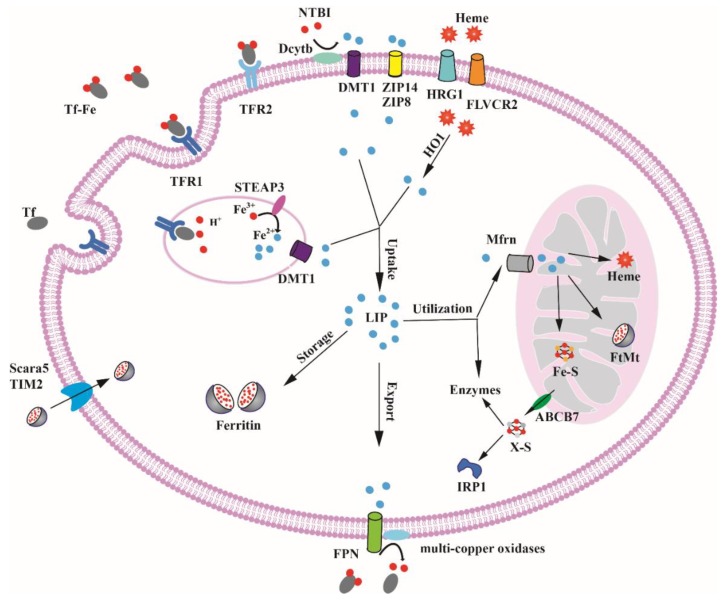
Iron metabolism in mammalian cells. Transferrin-bound ferric iron enters most cells via TFR1-mediated endocytosis. Iron is freed from transferrin and reduced to ferrous iron by STEAP3 in endosomes. Further, ferrous iron is transported across the endosomal membrane to the cytosol via DMT1; TFR1 and apotransferrin (apoTf) return to the cytomembrane for further cycles. NTBI enters cells also through DMT1 after reduced by Dcytb. Ferritin is also taken up by cells via Scara5 and TIM2. Other iron acquisition pathways in specific cell types via TFR2, ZIP8, ZIP14, HRG1 and FLVCR2 are symbolized. The acquired iron enters into the cytosolic LIP. Then, iron is utilized for synthesizing iron proteins or transported to mitochondria via Mfrn, where the metal is inserted into heme and Fe-S clusters or stored in FtMt. The fraction of the LIP can be exported via ferroportin or stored in ferritin.

Iron is essential for cell, but excess iron is associated with diseases including liver disease, heart failure, diabetes, and cancer [6]. Hence, the regulation of iron uptake, storage, utilization and export is important for maintaining cellular iron homeostasis (Figure 1).

### 2.1. Cellular Iron Uptake

Iron released from duodenal enterocytes is delivered to tissues and organs by plasma transferrin (Tf) [7]. Tf contains two high-affinity ferric binding sites and about 30% binds with iron under physiological conditions, which is a crucial source of iron for mammalian cells [8]. Transferrin receptor 1 (TFR1) is a broadly expressed receptor which is used for delivering iron into cells from circulating Tf [9]. The Tf-Fe2/TFR1 complex is internalized via endocytosis and iron is released from the complex as a result of acidic environment in the endocytic vesicles. Free ferric ion is then reduced to the ferrous form by six-transmembrane epithelial antigen of prostate 3 (STEAP3) and transported into the cytosolic labile iron pool (LIP) via divalent metal transporter 1 (DMT1) [10,11]. Subsequently, TFR1 and apotransferrin (apoTf) are recycled back to the cell surface where Tf dissociates from TFR1 and apoTf is thus released [12].

Transferrin receptor 2 (TFR2) is the homolog of TFR1, and the amino acid sequence of TFR2 is 45% similar with that of TFR1 [13]. TFR2 is highly expressed in erythroblasts and hepatocytes have a lower affinity for Tf than TFR1 and are not important for iron uptake [14,15]. As a component of the iron-sensing mechanism, TFR2 contributes to hepcidin regulation in the hepatocytes and likely associates with erythropoietin receptor in erythroid cells [16,17].

There are several other Tf-independent mechanisms that mediate iron uptake. Non-transferrin-bound iron (NTBI) which has been identified in the plasma of patients with various pathological conditions dominated by iron overload could also be absorbed by cells [18]. NTBI is first reduced to ferrous form by the ferrireductase duodenal cytochrome b (Dcytb), and then transported into cells by divalent metal-ion transporter 1 (DMT1), Zrt- and Irt-like protein 14 (ZIP14) and Zrt- and Irt-like protein 8 (ZIP8) [19,20,21,22].

Iron bounded to proteins or small molecules can also be taken up by cells. T cell immunoglobulin-domain and mucin-domain protein 2 (TIM2) are receptors for H-ferritin (FTH1) and permit the cellular uptake of H-ferritin into endosomes [23,24]. TFR1 is an important cell-surface receptor for FTH1 in human cells which cannot express TIM2 [25]. Scavenger receptor class A, member 5 (Scara5) binds serum ferritin at the cell surface and then stimulates its endocytosis from the cell surface with consequent iron delivery [26].Fowler syndrome-associated protein 2 (FLVCR2) and heme response gene-1 (HRG-1) can internalize iron by import heme into the cells [27,28]. CD91 and CD163 mediate endocytosis of heme and hemoglobin arising from intravascular hemolysis are mostly expressed in macrophages [29,30], and heme oxygenase 1 (HO-1) catabolizes heme to biliverdin, carbon monoxide and free iron [31]. In summary, mammalian cells can acquire iron in various ways to satisfy the cellular iron demands in different physiological conditions.

### 2.2. Cellular Iron Utilization and Storage

Once it enters into the LIP, iron is readily available for utilization and storage. Iron in LIP is utilized for synthesizing iron proteins and enzymes in most cells. As a cofactor, iron is essential for enzyme activity. In the cytoplasm, poly(rC) binding protein1 (PCBP1) and its paralog PCBP2 bind iron and deliver it to iron-dependent prolyl hydroxylases (PHDs) and asparaginyl hydroxylase (FIH1) that regulates hypoxia-inducible factor (HIF) [32]; PCBPs can also enhance the incorporation of iron into the deoxyhypusine hydroxylase (DOHH) [33]. In the nucleus, ribonucleotide reductase (RNR) contains an oxygen-linked diferric center which catalyzes the reduction of ribonucleotides (NDPs) to deoxyribonucleotides (dNDPs) [34]. In addition to the LIP in cytoplasm, lysosomes also store a large amount of LIP. The lysosome LIP may be derived from the degradation of iron-containing protein in the cycle of intracellular iron [35].

In erythroid cells, most iron in the LIP is transported to the mitochondria, where it is assembled on heme and Fe-S clusters, and excess iron is stored in the form of mitochondrial ferritin. The transport pathways of iron toward mitochondria are still undefined, which may relate to transferrin-endosomes and gentisic acid [36,37]. Some researchers have reported the mechanism of iron influx into mitochondria. The accumulation of mitochondrial iron is tightly regulated by the expression levels of mitoferrins (Mfrns). Mfrn1 and Mfrn2 are homologous and contribute to mitochondrial iron delivery in many cell types. Mfrn1 is mainly expressed in developing erythroblasts [38]. Mfrn2 is ubiquitously expressed, however the overexpression of Mfrn2 cannot support hemoglobinization in erythroid cells deficient in Mfrn1 [39]. The synthesis of mitochondrial Fe-S cluster requires iron and cysteine as initial materials, and frataxin (FXN) which can bind ferrous iron plays an important and elusive role in this process [40]. Finally, two main components are synthesized by ISC machinery. Mitochondrial [4Fe-4S] clusters as a cofactor are trafficked to respiratory chain complexes and enzymes such as aconitase, and succinate dehydrogenase; another unknown sulfur-containing component X-S is delivered by ATP-Binding Cassette Subfamily B Member 7 (ABCB7) to the cytosolic ISC protein assembly (CIA) system for maturation of cytosolic enzymes and Fe-S proteins [41]. In non-erythroid cells, the synthesis of heme is carried out in mitochondrion and cytoplasm, respectively and regulated by the level of 5-aminolevulinic acid synthase1 (ALAS1), the first and rate-limiting enzyme of the heme biosynthetic pathway [42]. After synthesis, heme is transported out of the mitochondrion into the cytoplasm via the mitochondrial heme exporter FLVCR1b [43]. FLVCR1a is encoded by Flvcr1 gene just as FLVCR1b is expressed at the plasma membrane and controls the content of intracellular heme [44]. Mitochondrial ferritin (FtMt) is expressed in some tissues and its sequence is more similar to H-ferritin [45]. The function of FtMt has not been completely identified. Some results showed that the FtMt may protect mitochondria from iron-dependent oxidative damage and not involve in storing cellular iron [46].

In non-erythroid cells, the majority of LIP iron is incorporated into ferritin. Ferritin is a very important and ubiquitous iron storage protein that can contain up to 4500 iron atoms. Ferritin is composed of 24 subunits of heavy (FTH1) and light (FTL) ferritin chains, and the proportion of the ferritin subunits is different in various tissues and physiological conditions. Ferroxidase active sites are located on the FTH1, which can oxidate Fe^2+^ to Fe^3+^ and are essential for Fe^3+^ deposition into the core of ferritin, while FTL promotes iron nucleation and increases the rate of turnover of the ferroxidase activity. Cytosolic iron can be delivered to ferritin via the iron chaperone PCBP1 and PCBP2 in mammalian cell lines [47,48]. Ferritin can also participate in the regulation of cellular iron homeostasis by ferritin degradation and ferritin iron recycling. Ferritin is degraded in the lysosome and iron is released under iron deficiency conditions, while lysosomal targeting of ferritin in iron-replete cells did not involve autophagy [49]. Nuclear co-activator 4 (NCOA4), which delivers ferritin to lysosomes, is a selective cargo receptor for the autophagic turnover of ferritin [50,51,52]. The expression of NCOA4 is increased to mediate ferritin autophagy in iron-deprived cells. In contrast, NCOA4 is degraded to impede the autophagic degradation of ferritin under iron-replete conditions [53]. The deficiency of NCOA4 in a knockout mouse model led to iron accumulation in the liver and spleen [54].

### 2.3. Cellular Iron Export

The fraction of the LIP that is not utilized or stored can be exported through ferroportin (FPN) to maintain system iron homeostasis. As the major cellular iron exporter, FPN is highly expressed on the surface of enterocytes, macrophages, hepatocytes and placental cells [55]. The molecular mechanism of how iron is delivered to FPN has not been clarified. Recent studies found that iron chaperone PCBP2 binds with FPN and transports iron from DMT1 to FPN, and suppression of the PCBP2 expression decreases FPN-dependent iron export from cells [56,57]. The ferrous iron efflux from cytoplasm via FPN is oxidized to a ferric state by the multi-copper oxidases, thus allowing it to be bound with transferrin. Three mammalian multi-copper oxidases(ceruloplasmin, hephaestin, and zyklopen) have been reported to express in different types of cells [58]. Hepcidin is a peptide hormone secreted by hepatocytes that regulates dietary iron absorption and systemic iron homeostasis. Hepcidin binds to FPN and controls the concentration of FPN on the cell surface through promoting FPN internalization and degradation [59].

### 2.4. Cellular Iron Balance

Cellular iron levels are mainly balanced by the iron-responsive element/iron regulatory protein (IRE/IRP) regulatory system, and these proteins are involved in iron uptake, storage, utilization and export. IREs are 26–30 nucleotide long hairpin-forming sequences with a 5′-CAGUGH-3′ apical loop sequence [60]. IRP1 and IRP2 are two orthologous RNA-binding proteins which interact with conserved cis-regulatory hairpin structures known as IREs [61].

The interactions of IRE/IRP regulate the expression of the mRNAs encoding proteins for iron homeostasis. When intracellular iron levels are high, translational-type IRE−IRP interactions in the 5′ untranslated region (UTR) are disturbed resulting in the translation of the mRNAs encoding H- and L-ferritin, ALAS2, HIF-2α and FPN, which control iron storage, utilization, export, and hypoxic responses, respectively. Conversely, when intracellular iron levels are low, IRE−IRP interactions in the 3′ UTR stabilize the mRNAs encoding TFR1, DMT1 and CDC14A, which are related to iron uptake, iron transport, and the cell cycle, respectively. A recent study showed that profilin2 as a key regulator of membrane trafficking and endocytosis pathways is a novel IRP-interacting transcript which alters iron metabolism at the cellular level and affects body iron homeostasis [62]. In addition to intracellular iron levels, some iron-independent factors can also affect the IRPs binding activity, such as hypoxia, oxidative stress, hormones, viral infection, etc. [63]. Hypoxia can decrease the IRP1 binding to IRE elements and reduce IRP1 binding to HIF-2α activity, but increase IRP2 binding activity [64,65]. Under oxidative stress conditions, the activity of IRP1 and IRP2 is decreased to reduce intracellular iron levels and induce the expression of ferritin, which may be to prevent further oxidative damage [66,67]. As an iron-independent factor, estrogens can also regulate the expression of ferritin and TfR1 through affecting the IRP1 activity [68]. Viral infection may not directly affect the IRPs activity, but through other indirect factors, such as reactive oxygen species (ROS) [69,70].

IRP1 and IRP2 activity is controlled by different mechanisms in response to cellular iron homeostasis. Fe-S cluster, F-box and leucine-rich repeat protein 5 (FBXL5) are switches regulating the activity of IRP1 and IRP2, respectively. In human cells, a family with sequence similarity 96 member A (FAM96A) that delivers Fe-S cluster to apo-IRP1 regulates cellular iron homeostasis via IRP1 Fe-S cluster maturation and IRP2 stabilization [71]. The stability of FBXL5 is regulated by way of iron and oxygen reaction, depending on the presence of its N-terminal domain [72]. Under iron-replete conditions, IRP1 binds a Fe-S cluster to become a cytosolic aconitase enzyme, preventing IRP1 binding to IREs [73]; IRP2 interacts with the FBXL5 adaptor protein that recruits a SCF (SKP1-CUL1-F-box) E3 ligase complex, promoting IRP2 ubiquitination and subsequent degradation by the proteasome [74,75]. Under iron-deficient conditions, IRP1 loses the Fe-S cluster and enzyme activity, changing its conformation to bind the IRE [73]; the structure of FBXL5 becomes unstable, promoting IRP2 accumulation [72].

## 3. Alterations of Iron Homeostasis in Cancer Cells

**Figure 2:**
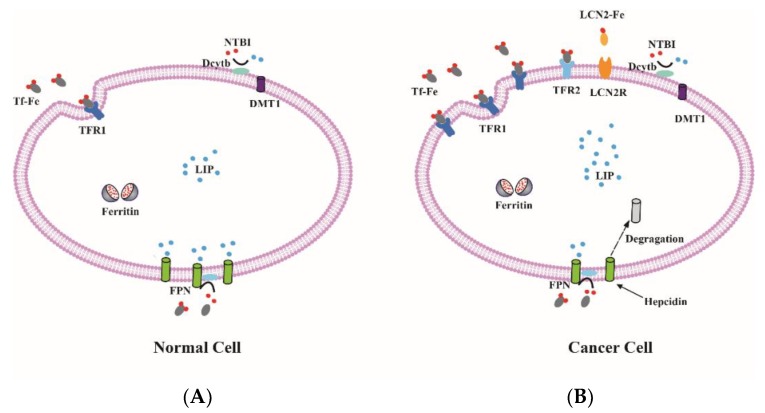
Iron metabolism in normal and cancer cell. (**A**) The expression of iron uptake proteins, such as TFR1, TFR2 and DMT1 are low, and the expression of iron export protein FPN is high in normal cells, resulting in a low level of labile iron; (**B**) By contrast, some cancer cells often have elevated TFR1, TFR2 and LCN2 as well as low FPN expression. Therefore, these cancer cells can increase intracellular iron to maintain the high demand for iron.

Compared with normal cells, cancer cells grow and proliferate rapidly and divide endlessly. These general features of cancer cells have a close relationship with iron (Figure 2). Iron plays a different role in cancer cells in different situations. As an electron donor for free radicals, iron can change cellular redox status. Excess free radicals contribute to gene mutation which may accelerate tumor initiation [6]. As a kind of nutrient element, iron is essential for cancer cell proliferation and DNA synthesis. Therefore, high iron requirement is a significant feature of many types of cancer cells (Table 1). The growing evidence indicates that cancer cells maintain the demand for high iron requirements by altering expression of iron metabolism-related genes and proteins.

### 3.1. MicroRNAs Regulated Iron Metabolism in Cancer

MicroRNAs (miRNAs) are small, non-coding RNAs that regulate gene expression by targeting complementary sequences in mRNAs, leading to mRNA degradation [102]. In recent years, many studies have demonstrated that miRNAs are also involved in the regulation of iron metabolism, including iron uptake, utilization, storage and export [103]. The regulation of iron metabolism by miRNAs may explain the alternations in cancer cells.

Based on the current literature, the expression of miRNAs always correlates negatively with iron uptake. miR-210 regulates the TFR expression level to maintain cancer cell proliferation and survival in a hypoxic environment [104]. The overexpression of miR-210 is correlates with good prognosis of cancer [105]. The expression of miR-152 is downregulated in human hepatocellular carcinoma tissue, which effectively inhibits the expression of TFR1 [106]. miR-Let-7d regulates the expression of DMT1-IRE and decreases iron accumulation in K562 cells [107]. In addition to regulating iron uptake, the expression of miRNAs also affects iron export. The expression of miR-20 may decrease iron export by targeting the mRNA of FPN in lung cancer [98]. miR-485-3p can also inhibit the expression of FPN under iron deficiency conditions [108]. miRNAs also control iron utilization and storage. miR-200b can increase the sensitivity of cancer cells to chemotherapy drugs by inhibiting FTH expression [93].

### 3.2. Altered Cellular Iron Uptake

As the primary way for iron to enter most cells, TFR1 is highly expressed in many cancer cells. TFR1 up regulation has been observed in a variety of cancer types including breast cancer, lung cancer, ovarian cancer, prostate cancer, pancreatic cancer and glioblastoma [109]. The expression of TFR1 affects cancer cell growth and proliferation. The c-Myc proto-oncogene could activate the expression of TFR1 to enhance cellular proliferation and tumorigenesis [110]. Mitochondrial respiration and ROS production are regulated by TFR1, which is essential for cancer cells growth and survival [111]. A recent study showed that epidermal growth factor receptor (EGFR), an oncogenic driver, maintains high level of cellular iron through regulating TFR1 distribution to modulate iron uptake [112]. TFR2 always expressed in erythroblasts and hepatocytes is also highly expressed in cancer cell lines, such as ovarian, colorectal, and glioblastoma [86]. However, the expression of TFR2 is inversely related to that of c-Myc and TFR1 [113].

DMT1 is highly expressed in colorectal cancer (CRC) through hypoxia-inducible factor 2a-dependent transcription, and iron uptake via DMT1 can promote colorectal tumorigenesis [88]. However, Dcytb that reduces NTBI to ferrous for iron absorbed through DMT1 does not affect intracellular iron in breast cancer cells [114]. However, Scara5 promotes cellular iron uptake by binding serum ferritin, but the overexpression of Scara5 significantly suppresses breast cancer cell proliferation by inactivating the extracellular signal-regulated kinase1/2 (ERK1/2), signal transducer and activator of transcription 3 (STAT3), and serine/threonine kinase AKT signaling pathways [115]. The overexpression of HO-1 promotes cancer cell proliferation and survival, and then promotes angiogenesis by up-regulating the expression of angiogenic factors [116]. There is no further study of the relationship between ZIP8, ZIP14 and cancer.

The human 6-transmembrane epithelial antigen of prostate (STEAP) family contains STEAP1, STEAP2, STEAP3, and STEAP4. They are important metallo reductases in metal metabolism. The expression of STEAP1 and STEAP2 is up-regulated in some cancer types, such as prostate, bladder, colon, pancreas, ovary, testis, breast, etc., but their clinical effects for cancer therapy are unclear [117].Under hypoferric condition, STEAP3 expression was up-regulated to maintain tumor growth [118].

Recent research has shown that the transferrin-independent iron transport mechanism existed in the tumor microenvironment, and these transport mechanisms were dependent on the key molecules of lipocalin2 (LCN2) and siderophores [119]. LCN2 is also up-regulated in some cancers, including breast, cervical and pancreatic cancer, and LCN2 expression correlates with increased invasiveness and poor prognosis [89,90,91,92]. The expression of LCN2 influences the prostate cancer cells growth and invasion via activating ERK signaling pathway [90,120]. LCN2 regulates hypoxia-inducible factor 1 (HIF-1) through signal-regulated kinase ERK, and the vascular endothelial growth factor (VEGF) mediated by HIF-1 regulates breast cancer angiogenesis [121].

### 3.3. Altered Cellular Iron Storage

Under normal physiologic conditions, excess iron will be stored in ferritin to prevent excessive production of ROS. However, both high and low expression level of ferritin have been reported in cancer cells [82,93]. Cancer cells may control the intracellular LIP to reduce oxidative stress and meet their rapid proliferation by altering the expression of ferritin. Ferritin which may derive from tumor associated macrophages is overexpressed in many cancer tissues, but not in cancer cells [122]. The expression of ferritin is low in human breast cancer cells with an epithelial phenotype; however, in breast cancer cells with an aggressive mesenchymal phenotype, the expression of ferritin is up-regulated [93]. A recent study indicated that FTH1 and FTL were expressed higher in glioblastoma compared with non-neoplastic brain [82]. Some inflammatory cytokines (IL-1β and TNFα) induce synthesis of FTH1, while the synthesis of FTL is mediated by nuclear factor-κB (NF-κB) signaling, which is responsive to oxidative stress [123,124]. Down-regulation of ferritin could disturb the tumor microenvironment and increase the sensitivity of cancer cells to chemotherapeutic drugs [125]. The proto-oncogene c-Myc could increases the LIP through repressing the expression of FTH1 and stimulating the expression of IRP2 [126].

### 3.4. Altered Cellular Iron Export

Cancer cells alter iron homeostasis not only by increasing iron uptake and controlling iron storage, but also by reducing iron export. FPN is the only known exporter channel for intracellular non-heme iron which is controlled by hepcidin. Different types of cancer exhibit altered regulation of FPN. FPN is down-regulated in breast cancer, lung cancer, ovarian cancer and prostate cancer [84,96,98,100]. The decreased expression of FPN leads to reducing the level of iron efflux and increasing the level of LIP in cancer cells. The added intracellular iron promotes cancer cells growth and proliferation. Nuclear factor erythroid 2-like 2 (NRF2) and myeloid zinc finger-1 (MZF-1) could affect cancer cells growth by regulating FPN expression in breast and prostate cancer [96,101,127]. Sirtuin 2 maintains cellular iron levels via deacetylation of NRF2 which leads to reduced FPN expression in cell lines and mice model [128]. Hepcidin synthesized by tumors or liver promotes FPN degradation, and also contributes to cancer growth and progression. Hepcidin is also synthesized in prostate epithelial cells, leading to an increase in prostate cancer cells and promoting prostate cancer cell survival [99]. The levels of serum hepcidin are increased in breast cancer patients, and impaired hepatic hepcidin expression could inhibit breast cancer growth in mice [96,97]. The multi-copper oxidases that oxidized ferrous iron to ferric state also have an impact on cancer cell growth by affecting intracellular iron metabolism. A recent study shows that up-regulated G9a, a H3K9 methyltransferase, could repress hephaestin and destruct cellular iron homeostasis, which leads to iron increase and promotes the progression of breast cancer progression [129].

### 3.5. Altered Cellular Iron Balance

The mechanism of iron regulatory proteins that regulate iron uptake, storage and export in cells is becoming much clearer, but the influence of iron regulation on cancer is less certain. In recent years, some studies have focused on the effect of iron regulatory proteins on cancer. Both IRP1 and IRP2 are over-expressed in breast cancer, but only the overexpression of IRP2 is associated with decreased FTH1 and increased TFR1, resulting in an increase in the LIP, which may contribute to poor prognosis of some breast cancer patients [79]. Hypoxia induces the expression of TFR1 and DMT1 in HepG2 cells; however, overexpression of IRP1 could reduce the stability of TfR1 and DMT1 mRNAs under hypoxia condition [130]. IRP2 is overexpressed in colorectal cancer compared to normal colonic mucosa which is associated with mutations in BRAF, and its expression is positively correlated with TFR1 expression [94]. IRP2-positive is also a marker of poor prognosis in lung cancer [95]. The overexpression of sirtuin 3 decreases TFR1 expression by repressing the IRP1 and inhibits pancreatic cancer cells proliferation, and iron and TFR1 expression are higher in the sirtuin 3 null mice pancreas. Furthermore, the expression of sirtuin 3 is negatively related to the expression of TFR1 in human pancreatic cancer [131]. As the most important proteins for cellular iron balance, the relationship between iron regulatory proteins and cancer requires further study.

## 4. Therapeutic Opportunities for Cancer Based on Altered Iron Metabolism

**Figure 3:**
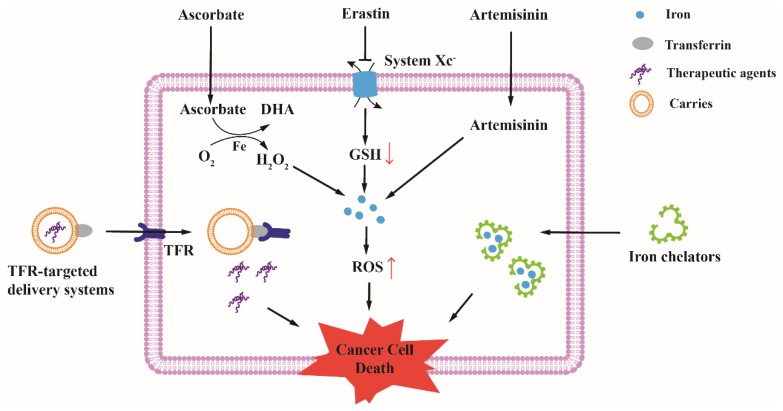
Therapeutic opportunities for cancer based on altered iron metabolism. TFR-targeted delivery systems can deliver drugs, proteins, nucleic acids, and viruses into cancer cells through binding to TFR, and these therapeutic agents induce cancer cell death through different mechanisms. Some potential anti-cancer drugs, such as Ascorbate, Erastin and Artemisinin, can disrupt redox homeostasis by intracellular high level of iron. H_2_O_2_ produced from high-dose ascorbate react with excess intracellular iron to generate ROS; Erastin deplete GSH (red ↑) by inhibiting system $X_{C}^{-}$, subsequently, excess intracellular iron lead to an increase of ROS levels (red ↓); Artemisinin react with excess intracellular iron to promote the production of ROS. Excess cytotoxic ROS induce cancer cell death. Iron chelators decrease intracellular iron by binding iron with a high affinity. Lack of iron in cancer cells inhibits cell growth and proliferation, leading to cell death.

As previously discussed, the demand of cancer cells for iron is higher than normal cells. The high intracellular iron level also indicates that the basal ROS levels are high in cancer cells, and thus cancer cells show more sensitive to the levels of iron and ROS. Hence, depleting intracellular iron, targeting abnormal proteins expression and disrupting intracellular redox homeostasis have been developed as efficient strategies for cancer therapy (Figure 3).

### 4.1. Depleting Intracellular Iron for Cancer Therapy

Iron chelators decrease intracellular iron level by binding iron with a high affinity. Deferoxamine (DFO) and Deferasirox (DFX) have been used for patients with iron overload. In view of important roles of iron in cancer cells, using iron chelators for cancer treatment shows great potential. Recently, novel iron chelator di-2-pyridylketone-4,4-dimethyl-3-hiosemicarbazone (Dp44mT) was also developed for cancer treatment. During the research processes, two different mechanisms of iron chelators have been explored [6]. The first one is that iron chelators inhibit the synthesis of enzymes and proteins by depleting intracellular iron. In addition, the second one is that iron chelators increase cytotoxic ROS in cancer cells by forming redox-active iron complexes.

DFO is an FDA-approved iron chelator always used for β-thalassemia, which has acquired favorable curative effect [132]. DFO inhibits proliferation and induces apoptosis by arresting the cell cycle, which appeared to be related to iron deprivation [133]. Many researchers have indicated that DFO has anti-cancer activity against some types of cancer cells. DFO has potent effects on human neuroblastoma cells, esophageal cancer cells and leukemic by inhibition of proliferation and differentiation [134,135,136]. In a clinical study, the overall response rate was 20% for hepatocellular carcinoma with DFO treatment [137].

DFX is an orally effective iron chelator and has a longer half-life than DFO in the plasma [138]. Researches have demonstrated that DFX could reduce cellular viability and proliferation in vitro, including small-cell lung cancer, oesophageal cancer and gastric cancer. Moreover, oral DFX was able to significantly suppress tumor growth in xenograft models [136,139,140]. Similar to the mechanism of DFO, DFX inhibits the cell cycle through reducing cellular iron acquisition and intracellular iron levels [136,139].

Several studies showed that Dp44mT inhibited many types of cancer growth in vitro and in vivo and was far more effective than DFO [141,142,143]. Unlike DFO, which just reduced iron, Dp44mT not only depleted cellular iron, but also formed Dp44mT-iron complex, which induced hydroxyl radical formation and increased ROS level in cancer cells [144,145]. This double mechanism is important for efficient anti-tumor effects.

As with cancer, neurodegenerative diseases also show an increase in intracellular iron levels. However, many clinical trials have failed to show any benefits of the use of these chelators in these diseases [146]. Compared to the efficacy of iron chelators for cancer, some clinical evidence shows that iron chelators also have obvious toxic side effects [147,148]. A recent review also showed concerns about clinical efficacy and side effects of iron chelators for iron storage diseases [149]. Although depleting intracellular iron is a promising therapy for cancer, iron chelation on cancer therapy needs more clinical evidence and new classes of iron chelators with fewer side effects, which needs to be developed in the future.

### 4.2. Targeting Iron Metabolism-Related Proteins for Cancer Therapy

Development of the therapeutic agents is based on characteristic markers of cancer, and these agents can selectively inhibit a specific target that is different in cancer cells compared with normal cells [150]. Due to differences in the expression of iron metabolism-related proteins between normal and cancer cells, the proteins are attractive targets for cancer treatment.

As a receptor that is highly expressed in a lot of cancer cells, TFR is a preferred target for cancer therapy [109]. Various TFR-targeted delivery systems have been developed, which consists of targeting ligands, carriers, and therapeutic agents.

Many ligands for targeting TFR have been studied, mainly including Tf, monoclonal antibodies, and single-chain antibody fragment (scFv). Tf is a natural ligand which can bind to TFR actively, which is first to be used for TFR-targeted ligands. Subsequently, monoclonal antibodies and scFv have been used for targeting the TFR. Monoclonal antibodies present the advantage of binding to an extracellular domain for the TFR that is different from the Tf-binding site and is, therefore, independent from Tf binding [151]. In comparison with the monoclonal antibodies or Tf, the scFv has a complete targeting ability and a much smaller size with better penetration into solid tumors [152]. In addition to the Tf, monoclonal antibodies and scFv, peptides can also be used for targeting the TFR [153].

There are two main strategies for linking targeting ligands to therapeutic agents. Previous strategies mainly using these ligands directly linked to some therapeutic agents; currently, some carriers have been developed as intermediates for linking ligands and therapeutic agents, mainly including liposomes, dendrimers, and nanoparticles. These carriers not only improve efficacy and safety in therapeutic agent delivery based on the EPR effect, but also result in an effective tissue distribution and prolonged half-life in tumor issue [154]. With the development of technology, many novel TFR-targeted delivery systems have been developed; more and more therapeutic agents can be loaded into delivery systems, such as drugs, proteins, nucleic acids, and viruses [109]. These agents can cause cytotoxicity or change the expression of proteins to induce cancer cell death.

Although many TFR-targeted delivery systems are still in the laboratory research stage, some have already entered clinical trials. MBP-426, which has recently entered a clinical II study, is a Tf-conjugated *N*-glutaryl phosphatidylethanolamine (NGPE)-liposome developed to improve the safety and efficacy of delivery of oxaliplatin into cancer cells by targeting TFR [155]. SGT-53 and SGT-94, targeting to TFR on cancer cells via a scFv to deliver the plasmid DNA into cells, are currently under clinical development [156].

In addition to TFR, methods targeting other highly expressed iron metabolism-related proteins also have been explored. LCN2, a potential diagnostic biomarker for breast cancer, is also a promising therapeutic target. When LCN2 was knocked down by ICAM-LCN2-LPs in breast cancer cells, VEGF and angiogenesis all reduced both in vitro and vivo [157]. Archazolid, a V-ATPase inhibitor, is highly effective against various cancer cell lines [158]. Recent research showed that Archazolid induced cell death by disturbing TFR recycling and impairing iron supply of cancer cells [159,160].

Ferritin, the natural protein cage, has been used as a nano-carrier for targeted delivery of anticancer drugs [161]. This ferritin-based delivery system is superior to inorganic nanoparticles as it is natural and biocompatible [162]. A recent study showed that ferritin could deliver curcumin into human breast cancer cell MCF-7 over-expressed ferritin receptors Scara5 and TFR1 [163]. Moreover, targeting down-regulation of FTL protein by the microRNA increased sensitivity of breast cancer cells to chemotherapy drugs doxorubicin and cisplatin [164]. Depending on the differences in protein expression, providing individualized therapy for different cancer patients will be a trend in the future.

### 4.3. Disrupting Redox Homeostasis by Intracellular High Level of Iron

Redox homeostasis is crucial for cell growth and proliferation, in which process iron and ROS play pivotal roles. ROS are a group of reactive chemical species that include superoxide anion (O_2_^−^), hydrogen peroxide (H_2_O_2_), and the hydroxyl radical (OH^•^). O_2_^−^ and H_2_O_2_ are mainly produced by respiration, and OH^•^ is mainly produced by the reaction of ferrous iron with H_2_O_2_ (Fenton reaction) [165]. Because of the higher electrode potential, OH^•^ is more harmful than O_2_^−^ and H_2_O_2_. Excess intracellular iron has a strong influence on the oxidative stress, because OH^•^ not only attacks lipids and proteins but also causes oxidative damage to DNA [166]. In cells, both iron and ROS are tightly controlled to keep redox homeostasis. Once the balance is broken, the fate of cell is death. Therefore, disrupting the redox homeostasis by intracellular high level of iron is a good choice for cancer therapy.

Ferroptosis is a recently discovered manner of cell death, whose main feature is the iron-dependent accumulation of lipid hydroperoxides [167]. It has been shown that the intracellular iron and ROS are the dominative initiators of ferroptosis, and glutathione peroxidase 4 (Gpx4) is one of the key regulators [168,169]. Recently, induction of the ferroptosis process by high level of iron in cancer cells has become an attractive method for redox-based therapy. Small molecules, such as Erastin, and Sulfasalazine, depleted GSH by inhibiting system $X_{C}^{-}$, and subsequently excessive iron and ROS induced ferroptosis [168,170]; RSL3, another ferroptosis inducer, inhibited Gpx4 which caused accumulation of lipid hydroperoxides [169]. Sorafenib, a multikinase inhibitor, has been approved for cancer therapy. Some recent researches showed that Sorafenib could induce ferroptosis in several cancer cell lines [171,172,173,174], and the anti-cancer mechanisms are involved in blocking system $X_{C}^{-}$ activity resulting in GSH depletion and iron-dependent induction of oxidative stress [170]. Artemisinin a traditional Chinese medicine isolated from Artemisia annua, was originally used in the treatment of malaria. Previous studies demonstrated that Artemisinin reacts with iron to promote free radical production which leads to the death of malaria [175,176]. Providing a high level of iron in cancer cells, artemisinin is subsequently suggested to be used as cancer treatment [177]. The anti-cancer activities of Artemisinin and its derivatives were also associated with the presence of iron and ROS production [178,179]. Recently, several studies showed that Artemisinin and its derivatives could also induce ferroptosis in some types of cancer cells, but the specific mechanism remains unclear [180,181,182].

Vitamin C (L-ascorbate) is an essential nutrient involved in many physiological and biochemical processes. Unlike all plants and most animals, who can synthesize vitamin C from glucose, humans cannot synthesize this vitamin because of the lack of gene encoding L-gulonolactone oxidase [183]. Due to it being an excellent electron donor that reduces oxidizing agents such as free radicals and reactive oxygen species ROS in cells, most of the previous studies reveal the anti-oxidant effects of vitamin C [184]. When catalytic metal ions exists, vitamin C can also show pro-oxidant effects [185]. Chen et al. reported that the anti-cancer effect of pharmacological ascorbate is dependent on the action of ascorbate as a pro-drug for H_2_O_2_ generation, which involves catalytic metals [186]. Although the history of vitamin C used for cancer treatment is full of controversy, intravenous pharmacological doses of ascorbate has been proposed as a potential anti-cancer therapy in recent years [187,188,189,190]. The mechanisms of high-dose ascorbate-induced cell death are closely related to intracellular high levels of iron. H_2_O_2_ produced from high-dose ascorbate react with excess intracellular iron to generate more cytotoxic ROS; moreover, H_2_O_2_ increased the availability of LIP in cancer cells, partially by disrupting Fe-S clusters [189].

Hence, disrupting the redox homeostasis by intracellular high levels of iron to induce ferroptosis or using H_2_O_2_ produced from high-dose ascorbate interact with intracellular iron to induce cancer cell death may provide additional benefits for cancer therapy in the future.

## 5. Conclusions

In the past few years, remarkable progress has been made in knowing cellular iron metabolism and the differences of iron metabolism between different types of cells. Cellular iron metabolism is tightly regulated to maintain iron homeostasis in normal cells. Cancer cells require large amounts of iron to meet the needs of growth and proliferation, therefore the cancer phenotype generally has a relationship with high iron requirements. The alterations of iron metabolism-related proteins are also inevitable.

Regardless of the reduction of intracellular iron by iron chelators, targeting iron metabolism-related proteins for the delivery of drugs or disrupting the redox homeostasis by increasing intracellular iron and H_2_O_2_, all these studies suggest that altering iron metabolism is a feasible way for treatment of cancer. In view of the close relationship between iron and cancer, therapeutic strategies for cancer based on altered iron metabolism will provide more possibilities. Even though great progress has been made in the earlier research, the complex relationship between iron and cancer needs to be clarified. Efforts are still needed to develop more efficient strategies for cancer therapy based on iron metabolism.

## Figures and Tables

**Table 1:** Expression level iron metabolism-related proteins in cancer cell.

Protein	Expression Level	Type of Cancer	References
TFR1	High	Esophageal adenocarcinoma	[76]
	High	Breast cancer	[77,78,79]
	High	Colorectal cancer	[80,81]
	High	Glioblastoma	[82,83]
	High	Ovarian cancer	[84]
	High	Prostate cancer	[85]
TFR2	High	Colorectal cancer	[86]
	High	Glioblastoma	[86,87]
DMT1	High	Esophageal adenocarcinoma	[76]
	High	Colorectal cancer	[80,81,88]
LCN2	High	Breast cancer	[89]
	High	Cervical cancer	[90]
	High	Cholangiocarcinoma	[91]
	High	Pancreatic cancer	[92]
Dcytb	High	Esophageal adenocarcinoma	[76]
	High	Colorectal cancer	[81]
Ferritin (FTH1, FTL)	High	Esophageal adenocarcinoma	[76]
	High	Glioblastoma	[82]
	Low	Breast cancer	[77,78,79,93]
IRP1	High	Breast cancer	[79]
IRP2	High	Breast cancer	[79]
	High	Colorectal cancer	[94]
	High	Lung cancer	[95]
FPN	Low	Breast cancer	[96,97]
	Low	Lung cancer	[98]
	Low	Ovarian cancer	[84]
	Low	Prostate cancer	[99,100,101]
